# Correction to: MAGE‑C2/CT10 promotes growth and metastasis through upregulating c‑Myc expression in prostate cancer

**DOI:** 10.1007/s11010-020-03958-6

**Published:** 2020-12-10

**Authors:** Jun Qiu, Bei Yang

**Affiliations:** 1 Center of Clinical Laboratory, The First Affiliated Hospital of Soochow University, No. 899 Pinghai Road, Suzhou, 215000 Jiangsu China; 2 Department of Imaging, The Central Hospital of Wuhan, Tongji Medical College, Huazhong University of Science and Technology, No. 26 Shengli Street, Jiangan District, Wuhan, 430014 Hubei China

## Correction to: Molecular and Cellular Biochemistry 10.1007/s11010-020-03814-7

The article “MAGE-C2/CT10 promotes growth and metastasis through upregulating c-Myc expression in prostate cancer”, written by “Jun Qiu and Bei Yang”, was originally published electronically on the publisher's internet portal on 06 July 2020 without open access. With the author(s)' decision to opt for Open Choice the copyright of the article changed on 19 October 2020 to © The Author(s) 2020 and the article is forthwith distributed under a Creative Commons Attribution 4.0 International License (https://creativecommons.org/licenses/by/4.0/), which permits use, sharing, adaptation, distribution and reproduction in any medium or format, as long as you give appropriate credit to the original author(s) and the source, provide a link to the Creative Commons licence, and indicate if changes were made.

The images or other third party material in this article are included in the article's Creative Commons licence, unless indicated otherwise in a credit line to the material. If material is not included in the article's Creative Commons licence and your intended use is not permitted by statutory regulation or exceeds the permitted use, you will need to obtain permission directly from the copyright holder. To view a copy of this licence, visit https://creativecommons.org/licenses/by/4.0.

The original article has been corrected.

